# The ripple effects of offshoring in the United States: Boosting local productivity and capital investment

**DOI:** 10.1371/journal.pone.0284490

**Published:** 2023-04-20

**Authors:** Nattanicha Chairassamee, Oudom Hean

**Affiliations:** 1 Department of Economics, Kasetsart University, Chatuchak, Bangkok, Thailand; 2 College of Business and Challey Institute for Global Innovation and Growth, North Dakota State University, Fargo, ND, United States of America; Universiti Malaysia Sabah, MALAYSIA

## Abstract

We investigate the offshoring effect on local productivity, physical and intellectual capital investment at the U.S. county level from 1999 to 2006. By using regression with fixed effects and instrumental variable to account for possible endogeneity, we find that offshoring can increase overall local productivity and capital investment. Through industry linkages, an increase in productivity and capital investment from offshoring enhances those increases in non-offshoring industries. Industries in both MSA (urban) and non-MSA (rural) counties receive benefits of productivity expansion and capital investment from offshoring. The increased capital investment from offshoring could be a channel of local productivity and capital investment expansion.

## 1. Introduction

The public often blames offshoring for its local labor replacement. However, Grossman and Rossi-Hansberg [1] provide a theoretical framework that shows an indirect positive effect of offshoring on the labor market through productivity expansion. Yet few studies investigate the effect of offshoring on productivity.

The objective of this study is to examine the offshoring effect on local productivity expansion. We find that offshoring can significantly increase productivity at the county level. Additionally, we show that an increase in offshoring stimulates regional capital investment, which could be a channel of local productivity expansion. We also examine the heterogeneous effects of offshoring by industry and region. We show that offshoring has positive effects on productivity and capital investment of “offshoring” and “non-offshoring” industries. Finally, we generally find significant positive impacts of offshoring on these economic outcomes in both metro- and non-metropolitan areas.

To measure offshoring, we follow the seminal work of Freenstra and Hanson [2] which uses imported intermediate inputs. We examine overall changes in productivity in counties with different levels of offshoring, by virtue of differences in their pattern of industrial specialization. To account for endogeneity, we apply a shift-share instrument. We find a positive effect of offshoring on county’s productivity. One way that offshoring can increase productivity is by raising capital investment. We find evidence that offshoring increases regional capital investment including physical capital and intellectual capital. By raising capital investment, offshoring could increase output and productivity.

Our study also analyzes different effects of offshoring by industry and region. Through input-output linkages, offshoring could affect industries differently [3,4]. We find that offshoring positively affects the productivity and capital investment of “offshoring” and “non-offshoring” sectors. Additionally, offshoring varies across industries, which then differentially affects regions because of differences in the compositions of regional industries [5–7]. Although we do not find a significant impact of offshoring on metropolitan productivity, we find that offshoring can significantly raise capital investment in these regions. For non-metropolitan areas, offshoring increases both productivity and capital investment.

Our paper contributes to a growing literature on offshoring. The first contribution of our study is to explore both the offshoring effects on local productivity. Previous studies analyze the effects of offshoring on individual productivity [8], firms [9–11], industries [12,13], and country [14]. Offshoring itself likely varies across industries, which then differentially affects regions because of differences in the compositions of regional industries. In addition, firm heterogeneity in productivity could also be distributed unevenly across region [7]. Our paper, therefore, complements those studies by analyzing the effects of offshoring at the subnational level and provides information to local policymakers who are concerned with the productivity and economic growth of their regions.

Second, we provide evidence that offshoring can increase physical and intellectual capital investment, which could in turn raise productivity. Baum et al. [15] and Slaper [16] have both found that there is a positive relationship between offshoring and productivity growth. In other words, their research suggests that productivity tends to increase as offshoring increases. Offshoring improves firms’ technology by increasing equipment-labor ratio and R&D [17,18]. Offshoring also potentially yields positive effects on production by lowering a firm’s costs and raising profits, which allows firms to expand output [1,9–11,19,20].

The third contribution of this study is to investigate the heterogenous effects of offshoring by looking at industry linkages within an area. Acemoglu et. al. [3] and Pierce and Schott [4] show that offshoring can affect different industries differently, referred as reallocation or industry-linkage effect. Some papers, however, refer this effect as spillover effect. We also additionally consider the different offshoring effects on urban and rural areas.

The remainder of the paper is organized as follows. Section 2 outlines the conceptual framework that shows the impact of offshoring on local productivity and capital investment. Sections 3 and 4 describe the data and measurements used and the empirical methods, respectively. Section 5 provides the structure of the Shift-Share instrument and the estimation strategy. Sections 5 and 6 present empirical results, which demonstrate the findings of the overall and the heterogeneous effects by offshoring sector, respectively. Section 7 presents concluding thoughts.

## 2. Conceptual framework and hypotheses

Offshoring is defined as imported intermediate inputs/tasks that are used for production that could instead have been domestically produced internally within the same firm [21], and all suppliers and buyers remain in their respective locations [22]. Firms normally offshore less efficient parts of their production process by purchasing abroad those factors that can be produced more cheaply there than in the home country [9]. Offshore industries enjoy producing their goods with lower production costs, which leads to a net increase in total output, wages, and employment [1,20].

In this present study, we have three hypotheses:

*Hypothesis 1*: Offshoring has a significant positive impact on productivity and capital investment. According to Grossman and Rossi-Hansberg [1], offshoring can lead to increased productivity. One mechanism through which offshoring can boost productivity is by spurring capital investment. Another advantage of offshoring is that firms can lower their input costs. Lower marginal production costs resulting from offshoring can incentivize firms to expand their output, hire more high-skilled workers, and invest in complementary equipment capital [17]. In fact, offshore industries tend to be more capital-intensive and innovation-focused, as Lampert and Kim [18] have shown. Additionally, offshore industries tend to disproportionately employ high-skilled workers. Increased investment in machinery and equipment [23], human capital [24,25], innovation [26], and entrepreneurship capital [27] can lead to GDP and productivity growth.

**Fig 1:**
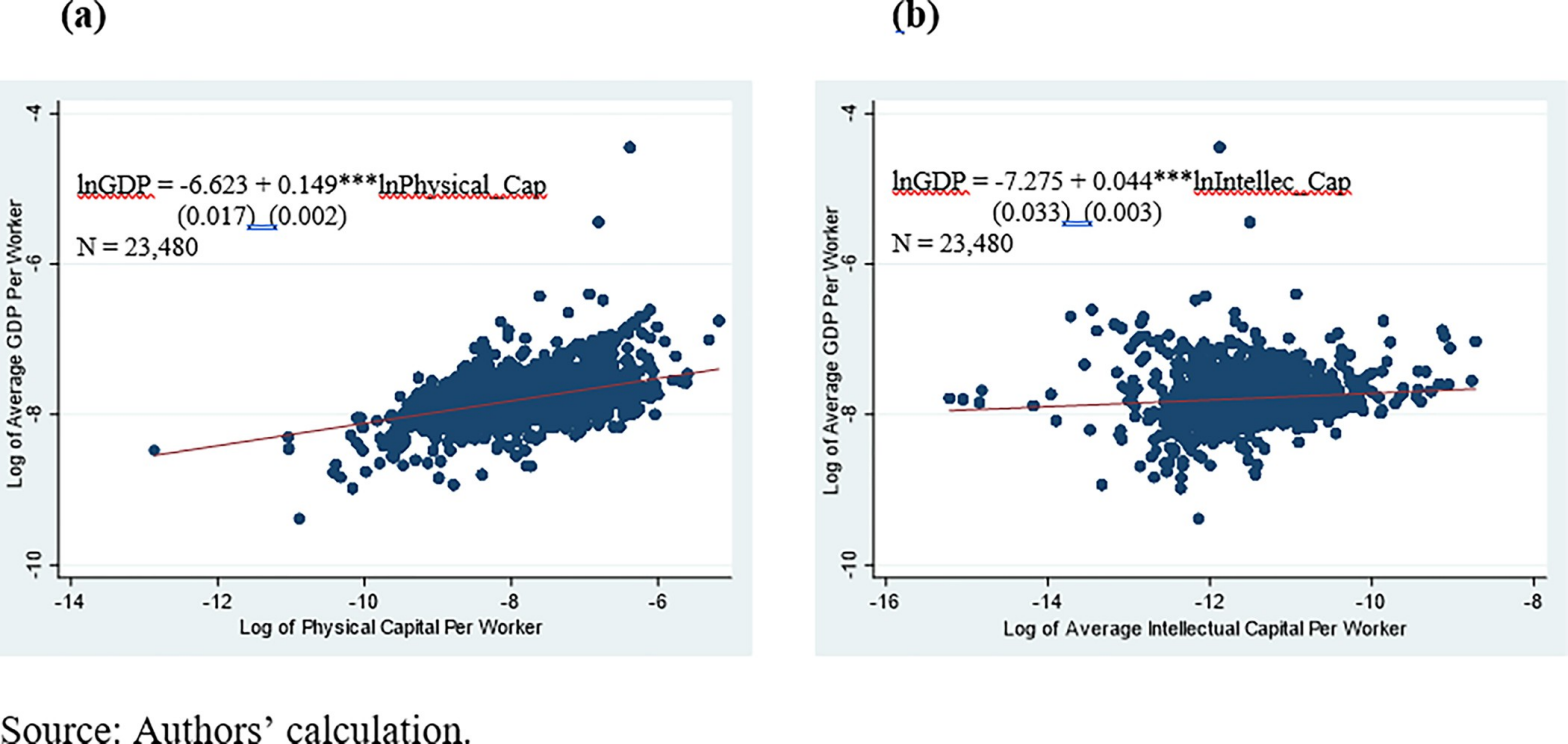
Relationships between productivity and capital investment. (a) Physical Capital and Productivity. (b) Intellectual Capital and Productivity.

Fig 1 shows the correlation between the average capital investment and productivity (measured by the total output per worker) using the U.S. county-level data from 1999 to 2006. As shown in Fig 1, both physical and intellectual capital investments are positively correlated to the productivity measure. Subsequently, we show that there is a positive relationship between offshoring and capital investment.

*Hypothesis 2*: Offshoring has varying impacts on productivity and capital investment across different regions. This is because international trade-related activities tend to vary across firms and industries, leading to differences in productivity levels even within the same industry or across different industries, as Bernard et al. [28] noted. These variations then translate into differential effects on regions due to differences in the compositions of regional industries. Moreover, variations in firm productivity levels can also be unevenly distributed across areas, as highlighted by Partridge et al. [7].
*Hypothesis 3*: The effects of offshoring on productivity and capital investment vary across different sectors. Offshoring is likely to have different impacts on productivity and capital investment depending on the specific characteristics of each sector. The offshoring of one industry could also affect other industries. Industries that are linked to offshore industries are likely to see changes in their output through the input-output matrix, otherwise referred to as the *industry input-output linkages* [4]. The effects of offshoring on employment and productivity of upstream and downstream industries are ambiguous [3]. The productivity expansion of an offshore industry may reduce its relative output prices [1]. For upstream effects, any domestic inputs that are used as complements to offshore inputs should grow as offshore productivity expands, which can stimulate the demand for inputs and increase employment. In contrast, offshoring can create a negative shock to the demand for given domestic substitution goods, which indirectly affects employment in industries that supply substitute inputs to the offshore industry. For downstream effects, an offshore industry may supply cheaper inputs and tend to induce downstream industries to consume these inputs. Therefore, offshoring can play an important role on productivity and capital investment within an area.

## 3. Empirical models

To measure the effects of offshoring on the regional productivity and capital investment, we estimate the following regression model:

$${logY}_{c,t}=\alpha_{0}+\alpha_{1}{logPOff}_{c,t}{+\alpha_{2}X_{c,t}+\pi }_{c}+\tau_{t}+\varepsilon_{c,t},$$ (1)

where *logY*_*c*,*t*_ is either the *log* of output per domestic worker or the *log* of capital investment per domestic worker in county *c* at time *t*. Specifically, capital investment is non-residential intellectual property products, which include software and research and development by BEA definition, and non-residential physical capital investment, which are structures and equipment.

*logPOff*_*c*,*t*_ represents the *log* of per-worker offshoring in county *c* at time *t*. Following Feenstra and Hanson [2], we measure offshoring by the total value of imported intermediates per domestic worker. *X*_*c*,*t*_ is control variables, which are total population, numbers of firms, and share of nonfarm employment. All control variables are in log form. We also include industry mix employment growth to capture any employment shocks that could affect productivity per worker. We calculate industry mix employment growth as follows: $Industry\;Mix\;Employment\;Growth_{i,t}=\sum_{i}Share_{i,c,t0}\Delta Emp_{i,us,t}$, where *Share*_*i*,*c*,*t*_ is the employment share of industry *i* in county *c* and in 1999, and Δ*Emp*_*i*,*us*,*t*_ is the national growth rate of industry *i* in year *t* relative to year 1999.

County- (*π*_*c*_) and year-fixed (*τ*_*t*_) effects are added to control for county-specific factors and any time-invariant factors.

### 3.1 Instrumental variable

The effects of offshoring in regression model (1) could be biased. Particularly, productive firms typically self-select to offshore, which leads to a reallocation of domestic labor and capital across firms and industries. To tackle potential endogeneity bias we construct a Shift-Share instrument for regional offshoring a`la Batik [29]:

$${IV}_{off,ct}=\sum_{i=1}^{n}\left(\left(\frac{{Off}_{i,c,t0}}{{Emp}_{c,t0}})∙(\frac{{Off}_{i,us,t}-{Off}_{i,us,t0}}{{Off}_{i,us,t0}}\right)\right).$$ (2)

The first term of this instrument is the per-worker offshoring in county *c* in the initial year 1999. Using the initial year’s shares reduces the potential for reverse causality between productivity and offshoring. It also shuts down variations in exposure to a change in the productivity or offshoring that might be driven by endogenous county responses, such as unobservable factors, which might affect firms’ decisions related to their production and offshoring. The second term of the instrument is the predicted national growth rates in industry *i*. To ensure the exogeneity (and to alleviate concerns about the spatial autocorrelation) of our instruments, we exclude the state wherein county *c* belongs, when calculating the national growth rates.

## 4. Data and measurements

Our data include 3,033 counties across the 48 contiguous states and the District of Columbia. To avoid the potential influence of major economic events, such as the Great Recession and subsequent trade-related shocks, including those stemming from President Trump’s trade policies, we have chosen to focus our study period on the years between 1999 and 2006.Our imported intermediates and domestic intermediate consumption are obtained from the Input-Output Table of the Bureau of Economic Analysis (BEA).

We use the BEA national Gross Domestic Product (GDP) and non-residential capital investment by industry to measure the output and capital investment of each county. County employment by industry is derived from the Bureau of Labor Statistics (BLS).

### 4.1 Productivity

We estimate local output by weighting the national real GDP by the employment share. Specifically, GDP in county *c* at time *t* is calculated as:

$${GDP}_{c,t}=\sum_{i=1}^{N}\left(\frac{{Emp}_{i,c,t}}{{Emp}_{USA,i,t}}∙{GDP}_{us,i,t}\right).$$ (3)

Here, *Emp*_*i*,*c*,*t*_ is a county *c*’s employment in period *t* for industry *i* and *Emp*_*us*,*i*,*t*_ is the US employment in industry *i* in period *t*. We use the 1997 North American Industry Classification System (NAICS), which includes data from the 3-digit subsector codes for 87 industries in both the manufacturing and non-manufacturing sectors. The first term in parenthesis, therefore, is a share of county *c*’s employment in industry *i*. The second term is national GDP by industry.

It is important to note that the BEA only provides the county GDP by industry from 2001 onward. However, our method of obtaining the local GDP offers two advantages. First, this method allows us to obtain county’s GDP before 2001. Second, national data offer details about industries. To verify the accuracy of our constructed-county GDP, we conducted a regression analysis by regressing the county GDP from the Bureau of Economic Analysis (BEA) on our constructed-county GDP. The resulting coefficient was 0.702 with the R-square of 0.978, indicating a strong relationship between our constructed-county GDP and the BEA county GDP. This cross-check provides further evidence supporting the reliability of our data.

Consequently, local per-worker productivity is obtained by dividing the county’s GDP by the county total employment.

$$productivity_{c,t}=\frac{GDP_{c,t}}{{Emp}_{c,t}},$$ (4)

where *GDP*_*c*,*t*_ is county c’s total output in dollar value in year t, and *Emp*_*c*,*t*_ is the county’s total employment in that year.

### 4.2 Capital investment

Similarly, the capital investment in county *c* at time *t* is calculated as:

$${Capital}_{c,t}=\sum_{i=1}^{N}\left(\frac{{Emp}_{i,c,t}}{{Emp}_{i,t}}∙{Capital}_{us,i,t}\right),$$ (5)

where *Capital*_*c*,*t*_ is capital investment per worker of county *c* in period *t*. The first term in parenthesis is a share of county *c*’s employment in industry *i*. *Capital*_*us*,*i*,*t*_ is the national capital investment in period *t* for industry *i*. Capital investment data are obtained from the Bureau of Economic Analysis (BEA). The non-residential intellectual property product includes software and research and development defined by the BEA. The non-residential physical capital investment includes structures and equipment.

Per-worker capital investment is then obtained by dividing the county’s capital investment by the county total employment. That is, ${Pcapital}_{c,t}=\frac{{Capital}_{c,t}}{{Emp}_{c,t}}$.

### 4.3 Offshoring

We follow the seminal work of Feenstra and Hanson [2] to measure offshoring, which is a purchase of inputs belonging to the same industry as that of the producing firms. The key idea of this measurement is that one cannot observe the firm’s ability to produce various inputs itself; however, we can observe transactions between the industry producing inputs and the industry importing those inputs. As Hummels et al. ([21], pp.987-988) indicate: “We are relatively confident that the auto industry is capable of producing auto parts itself but may choose to offshore production of auto parts. When we see the auto industry purchasing imported inputs from some other industry (textiles, glass, electronics), we are less confident that these represent inputs that could have been produced within the firm”.

Comparing Feenstra and Hanson [2] measurement to the offshorable job index [30,31] and routine task intensity [32], all measurements are somewhat similar. Our data shows that industries that imported intermediates are concentrated in the finance and insurance, information, and manufacturing industries, which is consistent with the offshorable job index. Goos et al. [33] also find that the offshorable job index and routine task intensity may be correlated. Offshoring can be measured with various datasets. For examples, Monarch et al. [34] and Kondo [35] use the Trade Adjustment Assistance (TAA) dataset to demonstrate offshoring- or foreign trade-related layoffs. Asquith et. al. [36] use the National Establishment Time Series (NETS) database to calculate job flows affected by international trade. Offshoring measurements, however, that can be computed by a publicly available dataset have two main approaches. The first approach uses imported intermediates. By using the definition of narrow offshoring, We can be confident at some point that it can represent offshoring. Another approach uses the employment of multinational enterprises (MNEs). This approach seems to have more advantages in terms of representing offshoring [21]. An important concern about using MNEs’ employment, however, is that firms headquartered in the United States have less vertical linkage than their affiliates [37,38]. Even though, by definition, MNEs’ employment can be an appropriate offshoring measurement, weak linkages across establishments within firms could lead to a potential measurement error in offshoring.

Following Feenstra and Hanson [2], the US offshoring in industry *i* is calculated as follows:

$${OFF}_{USA,i,t}=\frac{Imported\;intermediates\;purchased\;with\;inindustry\;i\;in\;year\;t}{Domestic\;intermediate\;consumption\;in\;industry\;i\;in\;year\;t}$$ (6)

Basically, the national-level offshoring of industry *i* is a share of imported intermediates that industry *i* purchases from the same broad industry classification as industry *i*.

Offshoring at county level is calculated by using industry-county employment share to weight the national-level offshoring as follows:

$${OFF}_{c,t}=\sum_{i=1}^{N}\left(\frac{{Emp}_{i,c,t}}{{Emp}_{USA,i,t}}∙{OFF}_{USA,i,t}\right)$$ (7)

Per-worker offshoring is derived from dividing the county’s offshoring by the county total employment. That is, ${Poff}_{c,t}=\frac{{OFF}_{c,t}}{{Emp}_{c,t}}$.

Measuring offshoring by using conventional gross trade data may result in double-counting due to multiple border crossings of goods and services [39]. Net value-added of trade, which requires bilateral trade data from multiple countries, can be used to address this issue. However, due to data limitations in our study, we rely on conventional gross trade data to measure offshoring. Offshoring measurement could also generate biases from the absence of accurate price deflators, resulting in biases to real value added and productivity statistics [40].

According to the aforementioned definitions of offshoring, there are 47 offshoring industries and 40 non-offshoring industries (i.e., *OFF*_*c*,*t*_ = 0). We list these industries in Tables S.1 and S.2 in S1 Appendix. Of these 47 industries, most are in the manufacturing, information, finance, and insurance industries. Non-offshoring industries, whose shares of imported intermediates equal zero, mostly include the service sector, such as the wholesale and retail trades, transportation, and warehousing. The summary statistics are in Table 1.

**Table 1:** Summary statistics of selected variables.

	Observations	Mean	SD.
Per-worker productivity	23,480	4,405.657	3,249.063
Per-worker offshoring	23,480	6.24x10^-8^	1.22 x10^-7^
Per-worker physical capital investment	23,480	5,516.995	4,731.280
Per-worker intellectual capital investment	23,480	124.522	114.544
**Variables in log form used in specification**			
Log per-worker productivity	23,480	0.677	1.867
Log per-worker offshoring	23,480	-17.527	1.670
Log per-worker physical capital investment	23,480	-7.771	0.762
Log per-worker intellectual capital investment	23,480	-11.488	0.607

**Note:** Per-worker productivity and capital investments are measured in US dollars. Per-worker offshoring is measured as a share of imported intermediates.

## 5. Empirical results

The first-stage results, shown in column (1) of Table 2, of the instrumental variable estimations are highly significant. The F-statistics from the first-stage of the equation is about 30. Column (2) of Table 2 shows the estimated effect of offshoring on productivity. On average, a 1 percent increase in offshoring can significantly increase productivity by about 1.1 percent. Our results are consistent with previous studies that also find the positive effect of offshoring on productivity at the individual level [8], the firm level [10,11,20,41], and the industry level [12].

**Table 2:** Offshoring effects on productivity and capital investment.

	First Stage: Offshoring (1)	Productivity (2)	Physical Capital (3)	Intellectual Capital (4)
Offshoring Instrument	-2.376***			
	(0.770)			
Offshoring		1.095***	0.783***	0.427***
		(0.243)	(0.162)	(0.111)
Controls	Yes	Yes	Yes	Yes
Fixed Effects	Yes	Yes	Yes	Yes
Observations	23,480	23,480	23,480	23,480
Cragg-Donald Wald F statistic	30.21			

**Notes:** All independent and dependent variables are shown in the table are in log form. Standard errors clustered at the county level are in parentheses

*** p ≤ 0.01

** p ≤ 0.05

and * p ≤ 0.10.

By increasing capital investment, offshoring could raise productivity. We run regression Eq (1) by using physical and intellectual capital investment as dependent variables and offshoring as the key explanatory variable. The results of this estimation are shown in Columns 3 and 4, Table 2.

We find that offshoring can stimulate both physical and intellectual capital investment. On average, an increase in 1 percent of offshoring is associated with an increase in physical and intellectual capital investment by 0.78 and 0.43 percent, respectively. These results support our first hypothesis that offshoring significantly increases productivity and capital investment.

## 6. Heterogeneous effects of offshoring

### 6.1 MSA and non-MSA counties

Offshoring might differentially affect productivity and capital investment of urban and rural regions. To examine our second hypothesis, which proposes that the effects of offshoring on productivity and capital investment differ across regions, we analyze the impacts of offshoring on both metropolitan statistical area (MSA) counties, which we refer to as urban/metro, and non-MSA counties, which we refer to as rural/nonmetropolitan. Table 3 shows the results of this analysis.

**Table 3:** Offshoring effects on productivity and capital investment by areas.

	Productivity (1)	Physical Capital (2)	Intellectual Capital (3)
**Panel A: MSA Counties**			
Offshoring	0.015	0.662***	0.314***
	(0.052)	(0.162)	(0.118)
Observations	7,526	7,526	7,526
**Panel B: Non-MSA Counties**			
Offshoring	0.253**	0.870***	0.502***
	(0.123)	(0.277)	(0.193)
Observations	15,954	15,954	15,954
Controls	Yes	Yes	Yes
Fixed Effects	Yes	Yes	Yes

**Notes:** All independent and dependent variables are shown in the table are dollar per-worker unit and in log form. Standard errors clustered at the county level are in parentheses

*** p ≤ 0.01

** p ≤ 0.05

and * p ≤ 0.10.

We find that offshoring does not exert any significance on productivity of the metropolitan areas. On other hand, a 1 percent increase in offshoring raises nonmetropolitan productivity by about 0.25 percent. In addition, the differential results are likely due to the different nature of industries and labor markets between metropolitan and non-metropolitan areas. Urban areas have more diverse industries [42].

For physical capital investment, a 1 percent increase in local offshoring increases this investment by 0.66 and 0.87 percent in MSA and non-MSA counties, respectively. Metropolitan intellectual capital investment increases by 0.31 percent when offshoring in these regions increases by 1 percent. Similarly, nonmetropolitan intellectual capital investment rises by 0.5 percent due to a 1 percent increase in local offshoring.

Promoting offshoring in a local area can directly affect productivity and capital investment. Offshoring can obviously benefit across sector to non-offshoring sector. The results suggest that offshoring is a source to enhance physical and intellectual capital investment, which could lead to local productivity expansion.

### 6.2 Offshore and Non-offshore sectors

Offshoring could have heterogeneous effects on different industries. Non-offshore industries that are linked to offshore industries are likely to see changes in their output and productivity through the input-output matrix [3,4].

To better understand the industry-linkage effects proposed in our third hypothesis, which says that offshoring has different effects on productivity and capital investment across different sectors, we estimate the effects of offshoring by industry. Specifically, we subdivide industries into two groups: offshoring and non-offshoring (offshoring index equals zero). Then, we estimate Eq (1) by the industry group.

As mentioned previously, non-offshoring industry is a group of industries that do not import any intermediates, while offshoring industry is a group of industries that import intermediates following the definition of narrow offshoring. Appendix contains the full list of these industries.

In Table 4, Panel A shows the results of offshoring sector. Offshoring has a significant positive impact on productivity and physical capital investment. An increase in 1 percent of offshoring stimulates productivity expansion and physical capital investment by 0.25 and 0.94 percent, respectively. Yet offshoring does not seem to have a significant impact on intellectual capital investment. Therefore, in the short run, physical investment could be easily accumulated more than intellectual capital investment since the latter is more related to human resources [43].

**Table 4:** Offshoring effects on productivity and capital investment by sectors.

	Productivity (1)	Physical Capital (2)	Intellectual Capital (3)
**Panel A: Offshoring Sector**			
Offshoring	0.248***	0.944***	0.129
	(0.095)	(0.172)	(0.094)
**Panel B: Non-Offshoring Sector**			
Offshoring	0.229***	1.712***	0.887***
	(0.086)	(0.286)	(0.184)
Controls	Yes	Yes	Yes
Fixed Effects	Yes	Yes	Yes
Observations	23,480	23,480	23,480

**Notes:** All independent and dependent variables are shown in the table are dollar per-worker unit and in log form. Standard errors clustered at the county level are in parentheses

*** p ≤ 0.01

** p ≤ 0.05

and * p ≤ 0.10.

In Panel B of Table 4, we find that offshoring significantly affects productivity in non-offshoring industries. A 1 percent increase in offshoring can raise productivity non-offshoring sector by 0.23 percent. This result suggests that there is a linkage between offshoring and non-offshoring industries. Non-offshoring industries could take advantages of either lower domestic inputs’ prices from offshoring industries or supplying complementary inputs to those offshoring industries to expand their production.

## 7. Discussion and conclusions

The role of offshoring on local productivity and capital investment is crucial in the labor market, yet its relationship has not received enough attention. To address this gap, our study empirically examines the effects of offshoring on U.S. counties’ productivity and capital investment.

Our study reveals that offshoring generally has a positive impact on local productivity, which can be attributed to an increased investment in physical and intellectual capital. The impact of offshoring varies across regions, with non-MSA (rural) counties showing better performance in capital investment compared to MSA (urban/metro) counties. These findings align with Lampert and Kim’s [18] research, which suggests that firms with lower R&D intensity levels benefit more from offshoring due to the pursuit of new knowledge. Therefore, it is likely that non-MSA counties, with their lower R&D intensity levels, are better suited for offshoring than MSA counties. Also, areas with a high intensity of offshoring activities may experience increased demand and supply of skilled labor, as regional innovation can increase local production of college graduates [44,45]. Our results may explain the insignificant negative effects (or decreased negative effects) of offshoring on U.S. employment in several previous studies [for example 8,20].

We also find that offshoring positively impacts productivity and capital investment in both offshore and non-offshore industries. The benefits spill over to non-offshore industries through input-output linkages, which has been documented in Pierce and Schott [4]. Offshoring has a significant impact on the labor market, affecting different types of workers such as low- and high-skilled workers, natives, and immigrants. The offshoring trend may have an unequal impact on low-skilled labor in offshore industries, forcing them to seek alternative employment in non-offshore industries where there is increased labor demand due to productivity expansion. However, it is also possible that low-skilled workers may complement the production process, and their skills could be improved through increased capital investment, as supported by studies such as Krusell et al. [46], Lewis [47], Parro [48], and Tyers and Yang [49]. Moreover, the local labor mobility across industries and sectors in the United States can also help to explain the offshoring phenomenon’s impact on the labor market. Therefore, offshoring can have complex and varied impacts on different types of workers, and understanding these impacts is essential for policymakers and businesses to make informed decisions about how to manage this trend’s effects on the labor market.

Our study finds that offshoring has positive effects on both productivity and capital investment, indicating that promoting offshoring activities can be a valuable tool for boosting local economic growth. Importantly, we also find that offshoring can have a disproportionately positive impact on rural areas, potentially helping to alleviate urban-rural disparities. These results highlight the potential benefits of offshoring for local economic development and underscore the importance of considering regional differences in any analysis of international trade activities.

To improve this research, several avenues can be explored. First, the spillover effects of offshoring across areas should be considered since industries in one area can use inputs from different industries in other areas. Second, different offshoring activities, such as material or service offshoring, could differently affect local productivity and capital investment. Third, this study’s results show average offshoring effects across sectors, and the specific effects on upstream and downstream across offshore and non-shore industries/sectors should be investigated (see [3,4,50] for cross-industry/sector backward and forward linkages). Fourth, this study’s data are mostly at the national level, which may generate measurement errors when examining offshoring effects at the county level. Moreover, the gross trade data used may suffer from double counting, leading to biased estimates. Fifth, this study only considers the intensity of industry offshoring at the county level. However, offshoring also depends on firm size, and larger firms are more likely to offshore [28,34], which can skew the results.

Finally, the COVID-19 pandemic may affect international trade-related activities and firms’ decisions to offshore. Therefore, policymakers should consider the pandemic’s impacts and design specific policies to support offshoring activities for productivity and capital investment expansion.

## Supporting information

S1 Appendix Lists of offshoring and Non-offshoring industries. (DOCX)
