# Supplementary material for: The ripple effects of offshoring in the United States: Boosting local productivity and capital investment
Source: PLoS One. 2023 Apr 20;18(4):e0284490. doi: 10.1371/journal.pone.0284490 (PMC10118088; doi:10.1371/journal.pone.0284490)
Supplement: S1 Appendix — (DOCX) [file pone.0284490.s001.docx]

**Appendix:** Lists of Offshoring and Non-Offshoring Industries

**Table S.1.** List of Offshoring Industries

| **NAICS** | **Description** | **NAICS** | **Description** |
| --- | --- | --- | --- |
| 111 | Crop Production | 112 | Animal Production |
| 113 | Forestry and Logging | 114 | Fishing, Hunting and Trapping |
| 115 | Support Activities for Agriculture and Forestry | 211 | Oil and Gas Extraction |
| 212 | Mining (except Oil and Gas) | 213 | Support Activities for Mining |
| 221 | Utilities | 311 | Food Manufacturing |
| 312 | Beverage and Tobacco Product Manufacturing | 313 | Textile Mills |
| 314 | Textile Product Mills | 315 | Apparel Manufacturing |
| 316 | Leather and Allied Product Manufacturing | 321 | Wood Product Manufacturing |
| 322 | Paper Manufacturing | 323 | Printing and Related Support Activities |
| 324 | Petroleum and Coal Products Manufacturing | 325 | Chemical Manufacturing |
| 326 | Plastics and Rubber Products Manufacturing | 327 | Nonmetallic Mineral Product Manufacturing |
| 331 | Primary Metal Manufacturing | 332 | Fabricated Metal Product Manufacturing |
| 333 | Machinery Manufacturing | 334 | Computer and Electronic Product Manufacturing |
| 335 | Electrical Equipment, Appliance, and Component Manufacturing | 336 | Transportation Equipment Manufacturing |

**Table S.1.** (Continued)

| **NAICS** | **Description** | **NAICS** | **Description** |
| --- | --- | --- | --- |
| 337 | Furniture and Related Product Manufacturing | 339 | Miscellaneous Manufacturing |
| 484 | Truck Transportation | 511 | Publishing Industries |
| 512 | Motion Picture and Sound Recording Industries | 513 | Broadcasting and Telecommunications |
| 514 | Information Services and Data Processing Services | 521 | Monetary Authorities-Central Bank |
| 522 | Credit Intermediation and Related Activities | 524 | Insurance Carriers and Related Activities |
| 541 | Professional, Scientific, and Technical Services | 561 | Administrative and Support Services |
| 562 | Waste Management and Remediation Services | 711 | Performing Arts, Spectator Sports, and Related Industries |
| 712 | Museums, Historical Sites, and Similar Institutions | 811 | Repair and Maintenance |
| 812 | Personal and Laundry Services | 813 | Religious, Grantmaking, Civic, Professional, and Similar Organizations |
| 814 | Private Households |  |  |

**Table S.2.** Lists of Non-Offshoring Industries

| **NAICS** | Description | **NAICS** | **Description** |
| --- | --- | --- | --- |
| 233 | Building, Developing, and General Contracting | 234 | Heavy Construction |
| 235 | Special Trade Contractors | 421 | Wholesale Trade, Durable Goods |
| 422 | Wholesale Trade, Nondurable Goods | 441 | Motor Vehicle and Parts Dealers |
| 442 | Furniture and Home Furnishings Stores | 443 | Electronics and Appliance Stores |
| 444 | Building Material and Garden Equipment and Supplies Dealers | 445 | Food and Beverage Stores |
| 446 | Health and Personal Care Stores | 447 | Gasoline Stations |
| 448 | Clothing and Clothing Accessories Stores | 451 | Sporting Goods, Hobby, Book, and Music Stores |
| 452 | General Merchandise Stores | 453 | Miscellaneous Store Retailers |
| 454 | Nonstore Retailers | 481 | Air Transportation |
| 482 | Rail Transportation | 483 | Water Transportation |
| 485 | Transit and Ground Passenger Transportation | 486 | Pipeline Transportation |
| 487 | Scenic and Sightseeing Transportation | 488 | Support Activities for Transportation |
| 492 | Couriers and Messengers | 493 | Warehousing and Storage |
| 523 | Securities, Commodity Contracts, and Other Financial Investments and Related Activities | 525 | Funds, Trusts, and Other Financial Vehicles |

**Table S.2.** (continued)

| **NAICS** | Description | **NAICS** | **Description** |
| --- | --- | --- | --- |
| 531 | Real Estate | 532 | Rental and Leasing Services |
| 533 | Lessors of Nonfinancial Intangible Assets (except Copyrighted Works) | 551 | Management of Companies and Enterprises |
| 611 | Educational Services | 621 | Ambulatory Health Care Services |
| 622 | Hospitals | 623 | Nursing and Residential Care Facilities |
| 624 | Social Assistance | 713 | Amusement, Gambling, and Recreation Industries |
| 721 | Accommodation | 722 | Food Services and Drinking Places |
